# “This Isn’t Just about Things, It’s about People and Their Future”: A Qualitative Analysis of the Working Conditions and Strains of Social Workers in Refugee and Homeless Aid

**DOI:** 10.3390/ijerph16203858

**Published:** 2019-10-12

**Authors:** Tanja Wirth, Janika Mette, Albert Nienhaus, Zita Schillmöller, Volker Harth, Stefanie Mache

**Affiliations:** 1 Competence Centre for Epidemiology and Health Services Research for Healthcare Professionals (CVcare), University Medical Centre Hamburg-Eppendorf, 20246 Hamburg, Germany; a.nienhaus@uke.de; 2 Institute for Occupational and Maritime Medicine, University Medical Centre Hamburg-Eppendorf, 20459 Hamburg, Germanyharth@uke.de (V.H.); s.mache@uke.de (S.M.); 3 Department of Occupational Medicine, Hazardous Substances and Public Health, Institution for Statutory Accident Insurance and Prevention in the Health and Welfare Services (BGW), 22089 Hamburg, Germany; 4 Department Health Sciences, Faculty of Life Sciences, Hamburg University of Applied Sciences, 21033 Hamburg, Germany; zita.schillmoeller@haw-hamburg.de

**Keywords:** job demands and resources, occupational health, social work, refugees, homeless, qualitative research

## Abstract

Large parts of Europe have been affected by an influx of refugees and increasing homelessness in recent years. Social workers provide care services for refugees and homeless people, but little is known about their working conditions. The aim of this study was to examine their job demands, resources and health strains. 26 semi-structured interviews were conducted with social workers in refugee and homeless aid in Hamburg and Berlin between October and December 2017. The interviews were analysed following Mayring’s qualitative content analysis. Additionally, the job demands and resources of social workers with and without long-term psychological strain were compared. Respondents particularly experienced demands concerning their job content and work organisation, including emotional and quantitative demands. Appreciation expressed by clients and social support from the team served as key resources. Respondents had problems switching off from work, were exhausted and exhibited signs of long-term psychological strain, such as symptoms of burnout or depressive states. Workers reporting long-term psychological strain were more likely to consider themselves as being adversely constrained by legal requirements and to describe inadequate supervision offers and team conflicts. In conclusion, the results indicate the need for job-specific health promotion measures reducing particularly demands concerning social workers’ job content and work organisation and further strengthening their social support.

## 1. Background

Large parts of Europe have faced considerable social challenges due to a large influx of refugees and increasing homelessness in recent years [1,2]. Refugees and homeless individuals are in especially vulnerable positions, with regard to their material situation, health and social integration [3,4]. Welfare services aim to support social development and social change in the population and help the disadvantaged to take action [5]. Accordingly, social workers provide refugees and homeless people with various advice and care services as well as emotional support. They help clients to integrate themselves into social structures and perform important networking and public relations work [3,6,7].

### 1.1. Immigration and Homelessness in Europe and Germany

The worldwide number of refugees has continuously increased over the past decade, e.g., driven by conflicts in Syria and the Middle East. Turkey hosted the highest number of refugees in Europe for the last five years [2]. In addition, Germany was the country with the world’s largest number of asylum applications in 2016 [8]. One of the reasons for this high level of immigration was an open-borders policy applied between September 2015 and March 2016 in Germany [9]. While the media and population initially encouraged a culture welcoming the refugees, a sense of mistrust began to develop from the start of 2016 [10,11]. The asylum policy was viewed more sceptically in the media, the concerns and fears of the population were brought into greater focus, and younger refugees were in some cases suspected of illegal conduct [11]. In the general population, more people in 2017 feared adverse effects caused by immigration than in 2015, such as strain on the welfare state (+15%) and an increase in social conflict (+9%) [10].

With the exception of Finland and Norway, the number of homeless people has increased in recent years in all countries of the European Union [1]. According to current estimates, around 650,000 people in Germany were homeless over the course of 2017 [12]. An annual analysis of the data from support institutions for the homeless indicated increasing diversity among homeless clients. For example, between 2007 and 2017, the proportion of clients who were female, single parents, couples with children, non-German or occupationally active, increased [13]. As with refugees, homeless people are also subject to some negative public attitudes. They are often stigmatised and discriminated against, and suffer from social isolation with little access to social resources [3,14]. This in turn can impede the efforts of workers in refugee and homeless aid institutions to represent the interests of their clients [7,15].

### 1.2. Working Conditions in Refugee and Homeless Aid

A recent literature review from 2019 shows that there has been little research conducted internationally or in Germany regarding the working conditions and health of social workers in refugee and homeless aid. On the whole, the review found that workers were exposed to high job demands, but also had various job and personal resources at their disposal [16].

Job demands of caregivers in refugee aid include experiencing the suffering of clients [17,18], communication problems and aggressive behaviour from clients, and low control at work due to legal regulations [18]. Workers may experience discrepancies in their job to help clients on the one hand and control them on the other. Their own as well as clients’ expectations may also not always correspond to actual available options, which to some extent are limited by structural or legal circumstances and constraints on resources [7].

According to a qualitative study in homeless aid, most factors that were perceived as work-related stressors by the workers also expressed their solidarity with the clients. This included sharing in their clients’ suffering and frustration about structural problems such as a welfare system that is perceived to be unfair [19]. Other identified factors were role conflicts [6] and a personal sense of responsibility felt by workers towards their clients, which in turn created difficulties in maintaining distance [15].

Job resources available to workers in refugee and homeless aid include a great sense of purpose in work and support from and exchange with colleagues [15,17,20]. Workers also considered acquired knowledge and skills to be a useful resource [17], as well as successful achievements with clients resulting from their work [15].

Studies relating to the health of workers show that a comparatively large proportion of workers in refugee and homeless aid exhibit secondary trauma [17], symptoms of post-traumatic stress disorder [21] and high levels of burnout [22]. A heavy workload, secondary traumatic stress, an unfavourable work environment and a diminished sense of achievement were associated with higher levels of burnout. Personal commitment and organisational support helped to mitigate the symptoms of burnout [22].

### 1.3. Theoretical Framework

The Job Demands-Resources model (JD-R model) from Bakker and Demerouti [23] served as a theoretical framework for this study. Due to its flexibility, the model can be applied to a diverse range of different professional and operational contexts. The model assumes that each occupation exhibits specific risk factors that can be classified into job demands and job resources. Job demands relate to aspects of the job that involve sustained psychological and/or physical effort and therefore incur psychological and/or physiological costs. Job resources relate to aspects of the job that can be helpful in the achievement of work goals, reduce job demands and support personal development. The model describes two processes, with job demands leading to exhaustion and health problems, whereas job resources serve to motivate, e.g., potentially resulting in high work engagement [23,24].

### 1.4. Study Aims and Research Questions

Based on the underlying assumption of Bakker and Demerouti [23] that any occupational activity exhibits its own job demands and job resources, the aim of this study was to identify the job demands and resources experienced by social workers in refugee and homeless aid and to describe their experience with strain. The study also served to generate hypotheses on possible factors associated with psychological strain experienced by workers in these fields, which could then be further examined using quantitative methods.

The study aimed to provide answers to the following research questions: (1) What job demands and resources do social workers in refugee and homeless aid experience? (2) Which health strains do these workers perceive?

Additionally, differences in the subjective experiences of job demands and resources between social workers reporting no psychological strain and those who did report long-term psychological strain were examined.

## 2. Materials and Methods

### 2.1. Study Design

As part of a qualitative exploratory study, 26 semi-structured interviews were conducted between October and December 2017. The qualitative method enables the description of complex social phenomena from the perspective of those affected. Incorporating the perspectives of the social workers and studying them in their natural environment allows for a comprehensive and detailed understanding of the phenomenon under study [25]. In this study the qualitative method provides a complex insight into the working conditions of social workers in refugee and homeless aid. A realist perspective was adopted by describing the experiences of the respondents and focusing on the semantic content of the data [26]. Qualitative content analysis was applied, which is now viewed as an independent method [27]. The criteria of the COREQ checklist were used in describing the study [28].

### 2.2. Study Population and Setting

The study included social workers in Hamburg and Berlin who primarily worked in the field of refugee or homeless aid, who directly provided support or advice to refugees and/or homeless people, and had served in this field for at least half a year. Volunteers and workers in administrative positions such as asylum application reviewers were not included due to differences in their activities and responsibilities.

Participants were recruited from institutions in the refugee and homeless aid sector. Purposeful sampling was applied to the selection of institutions by contacting walk-in and residential facilities from various supporting organisations. These were informed about the study by email and telephone. At participating institutions, the information about the study was forwarded to the employees, who would contact the study team directly if they were interested in participating. This second step was consistent with the approach of convenience sampling [29].

### 2.3. Data Collection

A semi-structured interview guideline was prepared on the basis of the theoretical background. In addition to sociodemographic information, information about professional qualifications and work duties, the guideline encompassed the following topics: demands at work, job resources, social support at the workplace and health strains (Table A1). Other topics included in the guideline were coping strategies, support in private life, support services and health promotion offers. These are published elsewhere. The guideline was tested in a pilot interview with a former worker from refugee aid and small updates were made accordingly. The pilot interview was not included in the analysis.

All interviews were conducted face-to-face within the workers’ institutions in Hamburg or Berlin during their working hours. The interviews were conducted by a health scientist or a psychologist serving as researchers in the field of occupational health and safety and occupational psychology. Both had prior experience with qualitative methods. The interviews took an average of 51 min (range: 27 to 86 min). Interviews were conducted until no new findings could be expected from further interviews and data saturation was achieved.

All interviews were recorded and transcribed literally in accordance with the transcription rules of Kuckartz [30]. The completed transcripts were not sent back to the interview participants for correction. Written field notes were made immediately after each interview.

### 2.4. Data Analysis

The data analysis was performed using qualitative structural content analysis in accordance with Mayring [31]. Key features of this analysis include the systematic and rule-guided approach and the development of the category system at the centre of the analysis process [31,32]. The main categories in the category system were created deductively based on the interview guideline. Job demands and resources were allocated to the categories of job content, work organisation, social relations, work environment and new forms of work [33]. Resultant health strains were categorised as psychological, physical/somatic and behavioural health consequences and as short-term and long-term consequences. Short-term consequences are immediate reactions to work-related stressors such as fatigue and stress that can be mitigated by means of rest. Long-term consequences on the other hand relate to chronic reactions such as health disorders [34,35]. Other sub-categories were derived inductively based on the data available. The encoding of the interviews was performed by the two interviewers. One interview was encoded by both researchers independently and the development of the sub-categories and allocation of text segments were compared. Where inconsistencies arose, a consensus was reached. All other interviews were encoded by one of the two researchers. Unclear text passages and codes were discussed jointly in regular meetings. The text segments allocated to the codes were paraphrased, generalised and subsequently reduced in accordance with Mayring [31]. The software MAXQDA 11 (VERBI GmbH, Berlin, Germany) was used to encode the data. 

In addition to this category-based perspective in data analysis, a case-based perspective was adopted, for which Kuckartz’s approach was selected [36]. Kuckartz recommends the creation of case summaries in connection with qualitative content analysis in which main and sub-categories can be presented for individual cases. These case summaries allow among other things a selection of cases for in-depth analysis and an analytical comparison of cases using tabular case lists [36,37]. Case summaries were prepared for all respondents for the main categories of job demands, job resources and health strains. The summaries of workers without resultant psychological strain and of those with long-term psychological strain were then included in a tabular case list for more in-depth analysis. This comparison was chosen because the two groups exhibit high contrast and mental disorders are the most common cause of work incapacity in this professional group [38]. Workers with long-term resultant psychological strain are those who described experiencing depressive states or burnout symptoms. Commencement of psychotherapy was also taken as an indicator of long-term resultant psychological strain. Quotes from the interviews were translated into English by a native speaker. Respondents are only provided with the study results in the form of a report once the study is completed.

### 2.5. Ethical Considerations

Prior to the interview, all participants signed a declaration of informed consent regarding the performance and recording of the interview. The Medical Ethics Committee of the Hamburg Medical Association provided professional legal and ethical advice for the study (PV5652).

## 3. Results

### 3.1. Characteristics of the Study Population

The characteristics of the 26 interviewees are listed in Table 1. The majority of the respondents were female (65%). Their age ranged from 26 to 64 years. 62% of the respondents had a bachelor’s degree or diploma in social work/social education work and 58% had worked for no more than three years in the field of homeless or refugee aid.

###  3.2. Working Conditions

#### 3.2.1. Job Content

Table 2 provides an overview of the job demands and resources relating to the job content.

(1) Job Demands

In the course of their work, social workers frequently gain insight into the personal backgrounds and histories of their clients. Many respondents reported experiencing emotional demands as a result of experiencing these personal stories. Respondents found client trauma, fear, stories of escape, despair, experiences of violence, suicide attempts, deaths and client relapses to be especially distressing. One respondent described finding it difficult for some time to deal with these emotional demands at the start of her job:

“The problems I heard about (...) from people’s stories, yes, I took them to heart for a while.”

*[#16, male career-changer, homeless aid]*

Around half of respondents were under stress due to a high workload, which they attributed in particular to the large number of different tasks. They believed that they had too many clients assigned to them, which left too little time for each client. Due to personnel absences, it was also necessary to take on additional work from other colleagues. Other respondents described the workload as a wave, alternating between stressful and quiet phases.

Other demands related to working directly with the clients. Language barriers made communication with clients more time-consuming, made it more difficult to understand their behaviour, and in some cases resulted in misunderstandings. Support in the form of interpreters was not always available. Instead, employees had to help themselves:

“It’s a challenge to speak to people with whom you have no common language. But you learn this. You learn to communicate using hands and feet, with motions and gestures.”

*[#4, female social worker, refugee aid]*

Many respondents viewed incidents of verbal and physical violence in the institutions, in the form of conflicts between clients but also between clients and workers, as especially demanding:

“We aren’t always welcomed with open arms. What I mean is that we are aggressively abused verbally, and there is also non-verbal aggression or violence against us (...).”

*[#8, male career-changer, homeless aid]*

Clients also had high or unrealistic expectations. For example, they believed that the workers were responsible for everything and expected immediate or personal help. Respondents struggled to distance themselves from such demands. When working with mentally ill clients suffering from depression or addiction disorders, adequate help was often more difficult to provide. Some respondents felt somewhat out of their depth. Poor reliability and commitment among clients was also deemed to be difficult, as well as a lack of appreciation. For some respondents, it was tiring to have to keep motivating the clients again and again. Some clients demonstrated less respect for female workers, for example by taking their statements less seriously.

Many workers believed that they had limited possibilities to find solutions for their clients’ problems and felt frustrated and helpless due to their poor chances of success. Respondents further described difficult conditions in the institutions such as bureaucracy and cost pressure, (financial) dependency on and poor cooperation with public authorities, social barriers for clients and frequently difficult conditions in the housing market:

“That’s where I also see things spiralling, or I feel helpless (...). I sometimes find that very sad and frustrating. I always say I wish I could open a drawer and pull out 500 apartments (...).”

*[#3, female social worker, homeless aid]*

For certain groups of clients, such as those with severe trauma or addiction disorders, respondents claimed that the support services on offer were inadequate. Some considered the offers to be limited by legal requirements in general, and especially for certain client groups, such as EU immigrants. Some respondents in refugee aid found the asylum system as a whole to be unfair. Other factors named by respondents as barriers to solutions and success in work were clients not accepting help, abandoning efforts or not properly cooperating, especially in homeless aid. Another difficulty perceived by the workers was in actually developing a trust-based relationship to the clients in the first place.

Respondents also experienced conflicts in their role in operating according to statutory and professional standards. They found it hard to make tough decisions such as abandoning support measures. They also believed that there was conflict between the underlying legal requirements, the requirements of the funding institutions, and the needs, wishes and rights of the clients.

Regarding direct duties, employees described the administrative and documentation work as extensive. This resulted in particular in time management problems, in being overwhelmed with work and also being underchallenged:

“Sometimes we act as secretaries as well (...). I would say that there’s a lack of developmental opportunity when it comes to the educational side of things.”

*[#14, female social worker, refugee aid]*

(2) Job Resources

In terms of job resources, almost all respondents reported that they experienced appreciation from clients for the work done, for example in the form of small gifts:

“I have met such nice people, (…) and the people bake something for you, or the children draw pictures. So you do get something back.”

*[#5, female social worker, refugee aid]*

Although many respondents reported the poor opportunities for solutions to be a burden, the majority of respondents also experienced success. Workers were motivated by the ability to have an impact on society, for example regarding opinions on homelessness. Experiences of success, such as the approval of a client’s asylum application, were particular cause for joy among workers in refugee aid. Respondents also described clients undergoing positive developments such as success in school or work, and were pleased when they themselves were able to achieve something on their clients’ behalf:

“We experience success when someone moves into their own apartment, and it is rewarding when you got to play a role in that.”

*[#16, male career-changer, homeless aid]*

Another resource was the pleasure of working with people. Respondents described interactions with clients as very positive, nice and respectful.

Despite having to multitask with a variety of duties, many respondents specifically enjoyed the diversity of their work. They highlighted the ability to perform diverse jobs, to work to solve a variety of problems for clients and to cooperate with a wide range of professions and clients as particularly positive aspects. 

Several workers were also granted considerable latitude in their ability to take action and make decisions, for example in providing clients with support and in organising their work.

Furthermore, respondents saw a great deal of purpose in their work:

“(…) because this isn’t just about things, it’s about people and their future (…)”

*[#2, female social worker, homeless aid]*

#### 3.2.2. Work Organisation

Table 3 lists the job demands and resources relating to work organisation.

(1) Job Demands

Many respondents criticised the staffing conditions of the institutions. They felt overwhelmed due to inadequate staffing and too many care cases per worker:

“This is actually the main reason why we work to and beyond our limits here, because we don’t have enough staff.”

*[#8, male career-changer, homeless aid]*

Respondents also stated that the institutions lacked specialists, such as those with specific language skills. As a result of the high demand for qualified staff during the sudden large refugee influx, respondents reported unqualified personnel also being hired. Respondents were sometimes working alone or with too few other workers. They felt overburdened in conflict situations with clients and did not believe that their safety was guaranteed. Other staffing problems arose as a result of high sickness absence and high turnover within a respondent’s own team or supporting organisation.

In terms of working hours, many respondents reported regularly or sometimes working overtime, though the chance to take time off as compensation later was usually possible. Some of the employees also worked shifts. For them, an irregular shift pattern, limited organisational opportunities and short-notice changes in the shift plan were seen as stressors. Long working hours and shifts resulted in workers experiencing work-life conflicts:

“And otherwise I spent a long time looking for a hobby, which is insanely difficult when you’re working shifts like this.”

*[#6, female educator, refugee aid]*

Many respondents reported time pressure at work, especially as a result of high workloads and the urgency of tasks. They felt overwhelmed by the time pressure and feared that the quality of their work might suffer as a result:

“We get a lot of emergencies (...). And the problem is that we don’t have the time available to address these emergencies appropriately.”

*[#11, female social worker, refugee aid]*

Another demand was frequent work disruptions, such as those caused by urgent tasks and unannounced clients. Some respondents had to spend all of their breaks in the institution; these breaks were then also disrupted by clients or telephone calls. This hindered effective recreation and prevented the workers from distancing themselves from their work.

Respondents also reported that their work with other offices such as support services or public authorities was a challenge. When applying to other support services on a client’s behalf, they found that these services were already overbooked and that waiting times for appointments were long. Financial dependency on public authorities such as aid funding associations was also seen as a negative, for example due to the pressure to achieve certain client numbers/occupancy rates. When working with public authorities and employment agencies, respondents reported poor availability, a lack of contact persons, long waiting times, a lack of transparency in decision-making and unrealistic demands made by these offices to clients, such as completing forms that were difficult to understand. Social workers thus viewed themselves to be in a difficult position:

“Sometimes you have the feeling that you have to fight the authorities to get anything done for those seeking help.”

*[#12, male educationalist, refugee aid]*

For several respondents, support offers such as group supervision or team meetings/case meetings with colleagues were personally of no help to them, because they saw no need for these offers, considered them to be time-consuming or considered them to reinforce existing conflicts within the team. Other respondents expressed criticism about having too few opportunities for discussion and supervision, case meetings or training opportunities.

Job insecurity was also a topic among respondents in refugee aid. Due to temporary contracts and frequent restructuring and downsizing by their employers as a result of variations in client numbers, respondents experienced frequent changes in their duties and teams and were subject to fears about their livelihoods.

(2) Job Resources

In many cases, the quoted job resources were the direct opposite of the quoted demands. In terms of working hours, respondents considered the ability to be compensated for overtime to be positive. They also mentioned flexible working hours, the ability to take unpaid leave and good scheduling coordination within the team. Another identified resource was involvement in the scheduling:

“Requests about working hours are respected. There are different shifts and you can say which shift you prefer and you usually always get it.”

*[#4, female social worker, refugee aid]*

In terms of work processes, respondents found reassurance in having properly defined cover and handover rules for duty, holiday leave and sick leave within the institution. Regular duty handovers also encouraged the exchange of information within the team:

“We also always have handovers in the evenings and mornings. So there’s always an exchange (...) where important things can be talked about.”

*[#18, female social education worker, homeless aid]*

Respondents perceived little time and work pressure when they had no target figures regarding consultations to fulfil or when the amount of paperwork was deemed to be relatively low. Workers also valued the ability to freely organise their breaks. Some respondents felt that their duties and roles were clearly defined thanks to a good job training process and the availability of process plans for client support. Individual respondents also felt that having team members with specific skill sets provided them with relief. For example, one team member frequently handled clients with special psychiatric problems. Respondents also highlighted good cooperation with committed personnel within the district authority and with client networks:

“I always find networks really important. They just provide relief and when they work well, there’s a lot to gain.”

*[#24, female remedial therapist, refugee aid]*

#### 3.2.3. Social Relations

Table 4 provides an overview of the job demands and resources connected with social relationships with colleagues and superiors.

(1) Job Demands

Overall, social workers described fewer demands concerning social relations. Several respondents experienced different perspectives and working methods within the team, for example regarding how pressure and sanctions are applied to clients and the level of support provided for clients. This resulted in inter-personal difficulties and conflicts within the team:

“Everyone works differently, everyone ticks differently and coming to some kind of compromise in that, (...) we actually need to be pulling together and working in similar ways, but often that doesn’t work. And that’s how conflict develops in the team.”

*[#14, female social worker, refugee aid]*

Individual respondents also reported disputes and gossiping within the team as well as performance pressure among colleagues. A high workload was one of the factors that respondents named as being responsible for this. Respondents also complained about receiving too little support from colleagues, not enough exchange within the team and poor cooperation between teams in the institution. One of the causes named for the poor support was the high turnover within the team.

Regarding cooperation with superiors, respondents complained in particular about the lack of support and the lack of transparency in decisions made by senior executives:

“At the management level, some internal procedures should be made more transparent. A lot is decided behind closed doors.”

*[#4, female social worker, refugee aid]*

In that regard, respondents also criticised the lack of appreciation shown by superiors and employers. Several respondents reported superiors showing little interest in the needs of the employees and, among other things, ignoring ideas and requests from employees.

(2) Job Resources

A good team atmosphere is an especially important job resource and reinforced a sense of enjoyment from work and a sense of well-being. Almost all respondents described a good relationship and strong sense of belonging within the team. Key factors contributing to this were prolonged collaboration among the team members, effective and regular discussions, open resolution of differences of opinion, close cooperation and joint efforts to overcome challenges:

“You’re not on your own with your work here, that’s the good thing. It’s a bit like a tight-knit community. Maybe in part because this work is so hard (...).”

*[#5, female social worker, refugee aid]*

Many respondents reported receiving professional advice from colleagues, e.g., in difficult situations or in case of work-related problems. In this connection, respondents identified good support from their team and described a culture in which questions were asked openly. One respondent even saw discussions with colleagues as a substitute for supervision.

Respondents felt that they were provided with support from superiors, e.g., in the form of help in crisis situations, ensuring that breaks were taken and supporting measures to reduce workloads. Respondents also considered it important and a relief to have superiors available to speak to when work-related questions and problems arise:

“(...) that we have a boss who is receptive to us and takes us seriously. That’s definitely worth a lot (…) and it also makes the work a bit easier.”

*[#2, female social worker, homeless aid]*

Many workers experienced appreciation expressed by colleagues and superiors. This appreciation was received in the form of positive feedback and by being asked for advice, being trusted or having their problems taken serious. Methods named for enabling expression of appreciation within the team were the annual staff appraisals or a “positive session” at the start of the team meeting, where all team members say what they currently find positive about the team or their work.

#### 3.2.4. Work Environment

With regard to the work environment, few job demands and resources on the whole were named by the respondents. These few are listed in Table 5.

(1) Job Demands 

In terms of the premises of the institution, several respondents believed that their work was hindered by the inappropriate accommodation of the clients, e.g., with the facilities being overfilled, clients being accommodated in container apartments or being housed in multi-bed rooms despite suffering from mental disorders. Workers then, e.g., had no group rooms available for providing support, and conflicts developed more readily between clients. Another problem concerned the organisation of rest breaks. In several cases, no break room was available, meaning that breaks had to be spent in offices, resulting in more frequent disruptions by clients:

“Because they see me coming back in here—from the toilet or from a meal—they follow me to tell me ‘I’ve still got this and that and the other!’ And I’m thinking, ‘I’m on my break!’ It gets so hard to shut your mind off from these things.”

*[#9, female career-changer, homeless aid]*

Two respondents from refugee aid also quoted inappropriate workplace organisation resulting from limited office space and multi-person offices. This made space availability tight with a lack of privacy in confidential meetings with clients:

“We also work in this container (...). Of course, I can eventually go home and get some rest, but still, you sometimes spend 40 h here and it’s difficult. It’s tight, you haven’t got peace and quiet to talk.”

*[#14, female social worker, refugee aid]*

In terms of work equipment, mainly respondents in refugee aid reported having inadequate computing resources. In some cases, laptops had to be used for work as there were too few computers or only shared computer workstations.

(2) Job Resources

In terms of the work environment, job resources include good workplace organisation and client accommodation as well as adequate work equipment. Several respondents expressed satisfaction regarding their workplace on the basis of their own workplace, the ability to work in large offices in groups of two or the ability to bring their dog to work. Two respondents in homeless aid expressed positive opinions about how accommodating clients in individual apartments in their facility meant that they had fewer conflicts between clients to resolve:

“Now they all have their own little kitchen and their own bathroom (...). I think this helps a lot in eliminating conflict in shared accommodation.”

*[#18, female social education worker, homeless aid]*

#### 3.2.5. New Forms of Work

Only a few job demands and no job resources were allocable to new forms of work.

(1) Job Demands

Three respondents in refugee aid described a dissolution of work/life boundaries as a result of work-related emails and calls as well as messages from clients on their private mobile phones, e.g., for arranging appointments. This resulted in a mixing of work and private life:

“That means that the boundary isn’t clear anymore and then you get a lot of them writing to you during the night.”

*[#25, male educator, refugee aid]*

One worker in homeless aid felt under stress as a result of the increasing email communication among colleagues and with superiors. She considered herself obligated to always answer these immediately and often lacked explanations that were available in direct communication.

### 3.3. Health Strains

Table 6 provides an overview of the short-term and long-term psychological, physical/somatic and behavioural health consequences for respondents.

Difficulty in switching off from work at home was named by respondents as a short-term psychological strain. The respondents found it especially hard to switch off when there was a heavy workload, when appointments or deadlines had to be met, in especially stressful situations such as violent incidents or the discovery of deceased clients, or when problems arose at work or with clients:

“And especially when things aren’t going well with a client, it’s difficult not to take that home with you, which is to say, I don’t always manage to.”

*[#2, female social worker, homeless aid]*

Other frequently named short-term psychological effects were temporary exhaustion or fatigue and the experience of stress. Two respondents had experienced burnout-type states as long-term consequences. They mainly considered high quantitative demands and inadequate staffing in the institutions to be responsible for this. Three respondents had already suffered from depressive states. For one respondent who recurrently had to deal with depression, the combination of client problems and additional private problems was the main trigger:

“(...) whenever I’m not entirely at ease with myself, I find it very difficult to go to work. In that case I also really struggle to get out of bed in the mornings, because (...) you deal with other people’s problems every day.”

*[#3, female social worker, homeless aid]*

Respondents also described physical/somatic effects. A frequently named issue was problems with sleeping. Most of them associated this directly with their work, naming the inability to switch off from work, the fates and personal stories of their clients, resulting in nightmares or stress, as well as the heavy workload, as causes:

“There are some things like I wake up at 4 or 5. And I think it has something to do with it all being very intensive, very dense. It’s a very hard-to-define stress phenomenon (…).”

*[#12, male educationalist, refugee aid]*

At the behavioural level, several respondents were more easily irritable due to the stress levels, e.g., in client meetings. As a long-term consequence, respondents experienced more frequent sickness and high levels of sickness absence among workers in their institution. Some attributed the absenteeism to a poor team climate or the heavy workload. Several respondents also noticed that they had become socially withdrawn. Firstly, they had no desire to engage in any further conversations after work as a result of the large amounts of social interaction in their working day. Secondly, they were sometimes too exhausted to engage in private activities such as meeting friends:

“(…) sometimes I’m so tired from work that I don’t have any energy left to do anything and then I have to cancel my plans.”

*[#6, female educator, refugee aid]*

### 3.4. Results of Case Comparisons

A total of twelve respondents were included in the tabular case list (Supplement Table S1). Five of them had reported no psychological strain in the interviews. Seven respondents had experienced resultant long-term psychological strain such as depressive states or burnout and/or had commenced psychotherapy in order to deal with occupational stress.

While almost all of the respondents in the group without psychological effects worked in homeless aid, most of the respondents in the group suffering from long-term resultant psychological strain worked in refugee aid. The majority of workers not experiencing strain were male and over the age of 50 years. The majority of workers experiencing long-term effects were female and under the age of 50. The group of workers not experiencing strain included three career-changers. By contrast, all workers in the other group were qualified as social worker/social education worker or educator/remedial therapist. In terms of professional experience, there was no relevant difference.

In both groups, several workers named certain job demands relating to their job content such as experiencing clients’ personal stories and having few opportunities for solving clients’ problems. However, workers suffering from long-term effects more frequently described having few opportunities for solutions as a result of structural conditions and legal requirements; for instance, they perceived legal requirements to be unfair. Workers in this group also widely described role conflicts, e.g., between legal requirements and their personal beliefs. The handling of exaggerated expectations among clients was held to be difficult by both workers not experiencing strain and those experiencing long-term psychological effects. Three workers suffering from strain effects also described their clients as demanding.

In terms of work organisation, employees in both groups considered cooperation with public authorities and other offices, inadequate staffing, working overtime and job insecurity to be demanding. Some workers suffering from resultant long-term psychological strain also considered their shift duty to be stressful, did not feel that they had received sufficient job training, and experienced frequent work disruptions. In this group, two respondents each also had inadequate supervision offers available, found these to be unhelpful or saw their work as insufficiently financially rewarded.

Demands regarding social relations, the work environment and new forms of work were almost exclusively named by workers suffering from resultant long-term psychological strain. This included different working methods and conflicts within the team, inadequate support from superiors, working in overfilled facilities, not enough computer workstations and a dissolution of work/life boundaries.

Regarding resources in relation to the job content, there were no relevant differences between respondents not experiencing strain and those suffering from resultant long-term psychological strain. They enjoyed their work and felt success in their work with clients, saw their work as diverse and felt appreciated by their clients.

In terms of work organisation, some workers not experiencing strain reported good break arrangements in their institution and the ability to exchange with colleagues through handovers to be resources. Two workers suffering from resultant long-term psychological strain did highlight flexibility in working hours.

All workers in the group not experiencing strain had good social relationships at work. They described a good team climate that lived through the exchange and support of colleagues as well as expressions of appreciation from colleagues and superiors. Workers suffering from resultant long-term strain also described a good team climate with support and appreciation from colleagues and superiors. However, beyond that, they mentioned difficulties and conflicts within the team.

## 4. Discussion

This is the first study applying qualitative methods to comprehensively examine the job demands and resources and the resultant strains experienced by social workers serving refugees and homeless people in Germany. A variety of job demands and resources were identified. Most demands related to the job content and the work organisation, including major emotional and quantitative demands, difficulties in working with clients, limited opportunities for solving client problems and inadequate staffing. Key resources related to the pleasure of working with clients, receiving appreciation from clients and social support from the team. Respondents frequently experienced stress, had problems switching off from work and felt tired and exhausted. Workers suffering from resultant long-term psychological effects worked primarily in refugee aid and, compared to those not experiencing strain, described job demands caused by restrictive legal requirements, demanding clients, inadequate supervision offers, conflicts within the team, unfavourable work environment and a dissolution of work/life boundaries.

### 4.1. Working Conditions and Resultant Strain Experienced by Social Workers in Refugee and Homeless Aid

Our results confirm that some factors examined in previous studies constitute relevant job demands and resources among social workers in refugee and homeless aid.

In this study, the experience of clients’ personal stories was a high emotional demand for respondents. Previous studies have already reported the considerable stress experienced by workers as a result of confronting the suffering and traumatic stories of refugees [18,39], identifying e.g., a relationship between a high level of traumatised clients and stress symptoms [40]. On the other hand, perception of clients’ suffering is also a factor that can result in greater identification with the organisation [20]. Workers should, however, be prepared to be faced with clients’ psychological problems. Some would like more support in this area to enable them to identify such problems and better help their clients [18]. Other demands identified in relation to the job content are consistent with previous study results, among them the heavy workload [22,41] and the handling of clients’ high expectations and aggressive behaviour [15,42]. The difficulty in establishing a relationship of trust with homeless clients was also described by employees working with homeless youth [15]. Language barriers and cultural difficulties were already apparent from previous studies relating to refugee aid [18,42] and, as expected, also occurred in this study. However, social workers in homeless aid also reported language and cultural problems. This was not specified in previous studies and is due to the rising share of homeless EU immigrants in Germany [13]. Another major issue among respondents was the limited opportunities for solving their clients’ problems, which some of them attributed to restrictions imposed by legal requirements. An earlier study in Germany had already described how refugee aid workers were limited in their scope to take action due to legal regulations [18]. Workers serving refugees and asylum seekers in countries such as the United Kingdom also believed that they were restricted by the law in how they were able to help clients [42].

Some respondents felt that they were not adequately supported with services such as professional supervision. This was especially the case among respondents suffering from resultant psychological strain. Supervision is an important tool in examining one’s own approach to work and stress, and can help to protect the health of social workers [43]. The study results might lead to the assumption that a lack of such support offers could even have a negative impact on worker health. 

With regard to the work environment, the respondents reported overfilled facilities, poor accommodation for clients and a lack of break rooms, among other factors. So far, stress caused by the work environment had barely been addressed for this professional group [16]. However, previous studies indicated a relationship between a poor work environment and burnout symptoms [22], and a relationship between a work environment characterised by limited space, few opportunities to find quiet or relaxation and a high noise level and a concentrated workload, which in turn resulted in high risk of burnout [44]. A new demand exhibited among respondents was the dissolution of the line between life and work, which as far as we know had not yet been described for this group. This factor should be adopted in future studies and examined further, as a dissolution of the work/life boundary resulting from the growth in mobile information and communication technology at work may become increasingly important.

This study can confirm that the worker’s own interest in the work, the sense of purpose of the job, success at work and appreciation expressed by clients are key resources regarding the job content for social workers in refugee and homeless aid [15,41,42]. This is consistent with the knowledge that among refugee aid workers a great motivating factor in the work is the ability to help fellow human beings [18]. In addition to working with clients, respondents especially found pleasure in working as part of a team and receiving considerable social support from their teams. Previous studies had already identified social support as a key job resource in this field of work [20,39]. The degree to which social support could also provide a buffer against adverse effects from job demands among social workers could be a subject for further studies. Our results also confirm the findings of a previous study which showed that high turnover can have an adverse impact on support and cooperation within a team [15]. It therefore seems to be particularly important to encourage continuity in the team composition.

In the interviews, social workers reported experiencing various resultant health strains also observed in other qualitative studies, such as the difficulty in switching off from work [15], a subjective feeling of stress [42,43] and a high level of sickness absence [41], although the latter could be caused by a multitude of factors relating to job and private circumstances. The long-term psychological effects named were depressive states and symptoms of burnout. Previous studies had already described a high prevalence of mental illness and burnout among this professional group [21,45]. The prevalence of depression, however, was not significantly different to that of the general population [18]. That said, conclusive information in available literature is still scarce and in some cases inconsistent [16]. Among workers in refugee and homeless aid, secondary trauma is also frequently observed due to being confronted with clients’ traumatic experiences [17,21,22]. In this connection, no findings are available in this study. However, some respondents did directly associate their resultant strains such as sleeping problems with the exposure to personal stories of their clients.

### 4.2. Potential Factors Associated with Resultant Psychological Strain

The JD-R model proved to be a suitable theoretical framework for this study. By implementing the model practically, qualitative exploration of the working conditions also helps to identify unexpected job demands and resources [23]. In this case little was previously known about the specific field of work. The study revealed a series of job demands and resources for this professional group that may have had a direct impact on their experiences with strain and motivation. Indeed, workers associated factors such as a heavy workload and emotional demands with resultant strains such as an inability to switch off from work, stress, exhaustion, burnout and sleeping problems. On the other hand, they were motivated in their work by the appreciation expressed by clients, by the sense of purpose of their work and by a good team atmosphere.

The supplementary case comparison provided further insight into factors that may play a particular role in the development of long-term resultant psychological strain. This includes restrictions imposed by legal requirements, experiencing clients as demanding, inadequate supervision offers, conflicts within the team, a lack of support from superiors, unfavourable work environment and a dissolution of work/life boundaries. Based on the results of the study and previous research, it can therefore be assumed that there is a positive relationship between quantitative and emotional demands, limited control at work caused by legal requirements, inadequate opportunities for supervision, a lack of social support and unfavourable work environment on the one hand and resultant psychological strain on the other. It can also be assumed that the appreciation of clients, a sense of purpose in work and social support provide motivation and are linked to job satisfaction and engagement.

The precise processes between these factors and their relationships with resultant strain could be studied in the future for this professional group using quantitative methods. It would be possible to incorporate other factors such as individual resources and coping strategies into such analyses. A task of further research would also be to develop specific workplace interventions to improve working conditions with social workers serving refugees and homeless people.

### 4.3. Implications for Practice

The results of this study allow some initial implications to be derived regarding the development of job-specific health promotion measures at a structural and behavioural level.

In terms of behavioural measures, some responsibility lies at the political or structural level to take action such as providing greater financial resources and adjusting carer-to-client ratios. However, the organisations themselves can take certain measures of their own. Where shift work is involved, the ability to plan effectively and the involvement of the workers in the organisation of shifts should be ensured [46]. Workers should also be granted adequate break times and rooms to enable them to relax and recover. Good job training and guidelines on client support may help to clarify duties and ensure consistency in work methods. In addition, the exchange among the team members should further be encouraged, e.g., through suitable quiet and break rooms, regular team meetings and organised collective case meetings. Constant reallocations of staff within the team should be avoided. In areas where workers have contact with clients outside of working hours, e.g., when arranging appointments, mobile phones issued by the worker’s employer could help to avoid the need to give out private mobile numbers to clients, thus enabling a clearer work/life separation. In general, workers’ availability should be restricted from a certain time of day.

Behavioural health promotion activities would be a useful addition to the aforementioned measures. To support workers in dealing with their emotional demands, difficult cases and aggressive clients, supervision offers should be provided in all institutions in the form of individual or group supervision provided by external supervisors or peers [43,47]. Needs-based training, e.g., in the form of de-escalation training, may also provide key assistance. Workers in social work should also attend to their own interests, for instance by making a habit of maintaining a personal distance from their clients’ problems and their work.

### 4.4. Strengths and Limitations

The study addresses a current topic and field of work that is becoming increasingly important due to developments in refugee and homelessness figures. The qualitative method has enabled extensive insights into this field of work. The inclusion of a variety of supporting organisations and types of institution involved in refugee aid and homeless aid has also allowed for a broad perspective of the field. The reliability of the encoding process was ensured thanks to the involvement of two researchers [25]. To improve the reliability of the results, the categories and their content were described extensively, and direct interview quotes were provided.

However, various limitations should be considered. It is important to note that workers may perceive stressful circumstances differently. It is possible for distortion to arise as a result of self-selection, e.g., workers signing up for the interviews specifically because they experience especially high demands or strains [48]. The study is also based on the subjective perspectives of the respondents, which makes it possible that workers may have responded to questions in a “socially acceptable” manner. Data transcripts, analyses or interpretations were not taken back to the study participants, which may limit the accuracy of the study results [25]. Transferability of the results to other time periods and countries is limited due to the unique situation in Germany at the time the study was conducted [49]. On the other hand, it may also provide a unique insight into the consequences of the large refugee influx on the working conditions of these social workers.

## 5. Conclusions

The study provides a deep insight into the working conditions and strains experienced by social workers in refugee and homeless aid. The results show that social work in this field is, on the one hand, a very challenging job with specific demands, especially concerning the job content and work organisation. On the other hand, it can be very fulfilling for the workers, e.g., in terms of appreciation and social support received. The respondents expressed a variety of negative health strains that indicate the need for job-specific health promotion measures. Initial recommendations and implications for such measures in institutions for refugee and homeless aid could be derived directly from the results of this study. Further research on the job demands, resources and strains of this professional group in the form of quantitative analyses and intervention studies is necessary. The present study provides the required basis.

## Figures and Tables

**Table 1:** Characteristics of study participants (*n* = 26).

Characteristics	*n*	%
Gender		
Female Male	17 9	65.4 34.6
Age (years)		
≤30 31–50 >50	6 12 8	23.1 46.2 30.8
Range	26–64 years	
Area of work		
Homeless aid Refugee aid	14 12	53.8 46.2
Type of facility		
Residential home Walk-in counselling centre Day care centre, outreach social work Initial registration centre	12 7 4 3	46.2 26.9 15.4 11.5
Professional qualification		
Social worker, social education worker (bachelor, diploma) Career-changer Educator, remedial therapist Educationalist (diploma)	16 6 3 1	61.5 23.1 11.5 3.8
Professional experience in field (years)		
≤3 4–10 >10	15 6 5	57.7 23.1 19.2
Range	8 months–37.5 years	
Working time		
Full-time (≥36 h) Part-time (<36 h)	20 6	76.9 23.1

**Table 2:** Job demands and resources relating to the job content.

Categories	Content
*Job demands*	
Emotional work	Personal stories of clients
Workload	High workload, too many clients, taking on duties of other personnel, fluctuating workload, low workload
Work with clients	Cultural and communication problems, experiences with violence, exaggerated expectations by clients, work with mentally ill clients, poor commitment/reliability, motivational work, role as man/woman, lack of appreciation by clients
Limited solutions for client problems	Due to structural conditions, due to legal requirements, lack of adequate support services, clients reject help
Role conflicts	Making difficult decisions, conflict: requirements and client representation, conflict: monitoring and helping clients, conflict: requirements and personal beliefs
Task-related demands	Paperwork and administrative workload, poor planning options, challenges in senior positions, monotonous tasks
Public perception	Prejudice against work/clients
*Job resources*	
Appreciation	Appreciation expressed by clients, appreciation from society
Experience of success	Social change, positive development of clients, positive news for clients
Work satisfies inclinations and interests	Enjoyment of work with people/client contact, positive duties, ability to apply interests
Diversity of work	Diverse fields of work and range of clients
Control at work	High control at work and decision latitudes
Purpose	Experience of purpose in work
Learning and development opportunities	New insights into cultures, countries and languages

**Table 3:** Job demands and resources relating to work organisation.

Categories	Content
*Job demands*	
Staffing problems	Staff availability, turnover, sickness absence
Working time	Overtime, shift work, work-life conflicts
Work processes	Time pressure, work disruption, organisation of rest breaks
Working with third parties	Working with other offices, working with public authorities
Inadequate support services	Difficulties in holding team meetings, organising supervision and conducting training activities
Job insecurity	Job insecurity caused by temporary employment, restructuring/downsizing
Financial security	Inadequate funding and unfair salaries
Poorly defined company profile/duties	Inadequate job training, poorly defined duties and services
*Job resources*	
Working time	Working hours well organised and applied
Work processes	Little work/time pressure; good rest breaks, cover and handovers well organised
Role clarity	Duties and roles well defined, defined authority within team
Networking	Working with other offices/networks
Financial security	Salary viewed positively
Job security	Job security in public service

**Table 4:** Job demands and resources relating to social relations.

Categories	Content
*Job demands*	
Cooperation within team	Different perspectives and working methods, conflicts/gossip, inadequate support/exchange, lack of organisational coordination
Cooperation with superiors	Difficulty in working with direct superiors, difficulty in contact with management level
Lack of appreciation	Lack of appreciation expressed by superiors, lack of appreciation expressed by colleagues
*Job resources*	
Social support	Good team atmosphere, friendly advice, support in team, support from superiors
Appreciation	Appreciation expressed by colleagues, appreciation expressed by superiors

**Table 5:** Job demands and resources relating to work environment.

Categories	Content
*Job demands*	
Premises of the institution	Inappropriate accommodation for clients, inadequate quiet/break rooms, inadequate workplace organisation
Work equipment	Inadequate work equipment
Environmental factors	High noise levels, unpleasant odours
Ergonomic design	Work involving long periods of sitting
*Job resources*	
Premises of the institution	Satisfaction with workplace organisation, accommodation of clients in individual apartments
Work equipment	Well-equipped

**Table 6:** Short-term and long-term strain experienced by respondents.

	Short-Term Strain	Long-Term Strain
Psychological	Difficulty in switching off from work, exhaustion/fatigue, stress, worry, anxiety, emotional turbulence	Depressive moods, burnout
Physical/somatic	Physical exhaustion, susceptibility to diseases, coughing/difficulty breathing	Sleeping disorders, headaches, musculoskeletal disorders, tinnitus, gastric disorders
Behavioural	Easily irritable	Absenteeism, social withdrawal, presenteeism, psychotherapy, unhealthy eating habits, loss of empathy

## Supplementary Materials

The following are available online at https://www.mdpi.com/1660-4601/16/20/3858/s1, Table S1: Case list of respondents not suffering from and respondents suffering from resultant psychological strain on multiple (>1) reported job demands and resources.

## Appendix A

**Table A1:** Extract of relevant topics and questions from the interview guideline.

Interview Topic	Interview Questions
Demands at work	(1) What challenges do you experience in your work with refugees/homeless people? (2) Do these stated challenges represent a burden for you? If “yes”, which ones in particular? a Do you remember certain situations from your working day that you perceived to be especially stressful? If “yes”, what were these? (3) Are there certain situations in which you felt overwhelmed or underchallenged by your work?
Job resources	(1) What do you like in particular about your work with refugees/homeless people? a Do you feel that appreciation is expressed for the work you do → by your clients? → by your colleagues? → by your superiors? b What do you think about the cooperation and work atmosphere within your team?
Social support at the workplace	(1) Do you receive support at your workplace if you have technical questions or need collegial advice? If so, from whom? a Do you have regular team meetings and/or meetings for supervision at your workplace? Are these helpful?
Health strains	(1) Do you notice (on yourself) that the demands you experience at work affect your health? If “yes”, what health impacts have you noticed? (2) Have there been certain situations in which you had the feeling that your work was negatively affecting your health? If “yes”, what kind of situations were these?
