# Supplementary material for: “This Isn’t Just about Things, It’s about People and Their Future”: A Qualitative Analysis of the Working Conditions and Strains of Social Workers in Refugee and Homeless Aid
Source: Int J Environ Res Public Health. 2019 Oct 12;16(20):3858. doi: 10.3390/ijerph16203858 (PMC6843662; doi:10.3390/ijerph16203858)
Supplement: Supplementary file 1 [file ijerph-16-03858-s001.pdf]

Table S1. Case list of respondents not suffering from and respondents suffering from resultant psychological strain on multiple (>1) reported job demands and resources

ID	Area of work	Gender, age (years)	Qualification	Professional experience (years)	Job content	Work organisation	Social relations	Work environment	New forms of work
Workers <u>without</u> resultant psychological strain									
#1	HA	F, 31–50	Career-changer	≤3	<u>Demands:</u><br>Emotional demands, heavy workload, exaggerated expectations of clients, motivational work, paperwork<br><u>Resources:</u><br>Appreciation expressed by clients, experience of success, pleasure in working with people/client contact	<u>Demands:</u><br>Staffing shortages, time pressure, cooperation with public authorities/other offices<br><u>Resources:</u><br>Good planning of break periods	<u>Resources:</u><br>Good team atmosphere, support and appreciation from team		
#17	HA	M, >50	Career-changer	4–10	<u>Demands:</u><br>Emotional demands, cultural/language barriers, experience of violence, limited solutions (clients reject help)<br><u>Resources:</u><br>Appreciation expressed by clients, pleasure in working with people/client contact	<u>Demands:</u><br>Being alone on duty<br><u>Resources:</u><br>Exchange through handover meetings	<u>Resources:</u><br>Good team atmosphere, support and appreciation from team, support and appreciation from superiors	<u>Demands:</u><br>Lack of quiet/break rooms	
#18	HA	F, >50	Social education worker	≤3	<u>Demands:</u><br>Limited solutions (structural conditions, clients reject help), role conflicts<br><u>Resources:</u><br>Appreciation expressed by clients, pleasure in working with people/client contact, diversity of work	<u>Demands:</u><br>Cooperation with public authorities/other offices<br><u>Resources:</u><br>Good planning of break periods, exchange through handover meetings	<u>Resources:</u><br>Good team atmosphere, support from team, support and appreciation from superiors	<u>Demands:</u><br>Work involving long periods of sitting	

ID	Area of work	Gender, age (years)	Qualifi-cation	Professional experience (years)	Job content	Work organisation	Social relations	Work environ-ment	New forms of work
#19	RA	M, >50	Career-changer	≤3	Demands:<br>Emotional demands, exaggerated expectations of clients, limited solutions (legal requirements), paperwork<br>Resources:<br>Appreciation expressed by clients, experience of success	Demands:<br>Staffing shortages, overtime, job insecurity	Resources:<br>Good team atmosphere, support from team, appreciation from superiors		
#23	HA	M, >50	Social education worker	>10	Demands:<br>Heavy workload, motivational work, experience of violence, limited solutions (lack of adequate services, clients reject help), poor planning options<br>Resources:<br>Appreciation expressed by clients, pleasure in working with people/client contact	Demands:<br>Staffing shortages	Resources:<br>Good team atmosphere, support and appreciation from team, appreciation from superiors		
Workers with long-term resultant psychological strain									
#3	HA	F, 31–50	Social worker	≤3	Demands:<br>Emotional demands, varying and heavy workload, exaggerated expectations of clients, limited solutions (structural conditions, clients reject help), role conflicts<br>Resources:<br>Appreciation expressed by clients, experience of success, pleasure in working with people/client contact, diversity of work	Demands:<br>Cooperation with public authorities/other offices, poor financial compensation<br>Resources:<br>Flexible working hours	Resources:<br>Good team atmosphere, support and appreciation from team, support from superiors		

ID	Area of work	Gender, age (years)	Qualifi-cation	Professional experience (years)	Job content	Work organisation	Social relations	Work environ-ment	New forms of work
#4	RA	F, ≤30	Social worker	≤3	Demands:<br>Emotional demands, heavy workload, too many clients, cultural/language barriers, demanding clients, limited solutions (legal requirements), role conflicts, poor planning options<br>Resources:<br>Experience of success	Demands:<br>Overtime, time pressure, work disruptions, cooperation with public authorities/other offices, supervision not viewed as helpful	Demands:<br>Different perspectives/work methods in team, inadequate support from superiors<br>Resources:<br>Good team atmosphere, support and appreciation from team		
#5	RA	F, 31–50	Social worker	≤3	Demands:<br>Emotional demands, heavy workload, too many clients, limited solutions (legal requirements, not enough adequate services), role conflicts<br>Resources:<br>Appreciation expressed by clients, experience of success, pleasure in working with people/client contact, diversity of work	Demands:<br>Staffing shortages, unqualified personnel, overtime, shift duty, cooperation with public authorities/other offices, supervision not viewed as helpful, poor financial compensation<br>Resources:<br>Flexible working hours	Resources:<br>Good team atmosphere, support from team, support and appreciation from superiors		Demands:<br>Dissolution of work/life boundaries
#6	RA	F, ≤30	Educator	≤3	Demands:<br>Emotional demands, cultural/language barriers, experience of violence, limited solutions to difficult cases, poor planning options	Demands:<br>Unqualified personnel, shift duty, cooperation with public authorities/other offices, inadequate supervision, job insecurity	Demands:<br>Performance pressure in team<br>Resources:<br>Good team atmosphere, support and appreciation		

ID	Area of work	Gender, age (years)	Qualifi-cation	Professional experience (years)	Job content	Work organisation	Social relations	Work environ-ment	New forms of work
					Resources:<br>Appreciation expressed by clients, experience of success, pleasure in working with people/client contact		from team, support from superiors		
#24	RA	F, ≤30	Remedial therapist	≤3	Demands:<br>Emotional demands, varying and heavy workload, too many clients, exaggerated expectations of clients, demanding clients, experience of violence<br>Resources:<br>Appreciation expressed by clients, pleasure in working with people/client contact, diversity of work	Demands:<br>Too few staff on duty, cooperation with public authorities/other offices, inadequate supervision, inadequate job training	Demands:<br>Conflicts in team, inadequate support from superiors<br>Resources:<br>Good team atmosphere, support and appreciation from team	Demands:<br>Overfilled facility	
#25	RA	M, 31–50	Educator	≤3	Demands:<br>Emotional demands, varying workload, exaggerated expectations of clients, demanding clients, limited solutions (structural conditions), role conflicts, paperwork<br>Resources:<br>Experience of success, pleasure in working with people/client contact	Demands:<br>Work disruptions, cooperation with public authorities/other offices, job insecurity, inadequate job training	Demands:<br>Different perspectives/work methods in team, conflicts/gossip, inadequate support and lack of organisational coordination in team, inadequate support from superiors<br>Resources:<br>Good team atmosphere, support from team	Demands:<br>Overfilled facility, too few PC worksta-tions, work involving long periods of sitting	Demands:<br>Dissolution of work/life boundaries

ID	Area of work	Gender, age (years)	Qualification	Professional experience (years)	Job content	Work organisation	Social relations	Work environment	New forms of work
#26	RA	F, >50	Social education worker	>10	Demands:<br>Heavy workload, experience of violence, motivational work, limited solutions (structural conditions, lack of adequate services), paperwork<br>Resources:<br>Appreciation expressed by clients, experience of success, pleasure in working with people/client contact, diversity of work	Demands:<br>Being alone on duty, shift duty, cooperation with public authorities/other offices, job insecurity	Demands:<br>Inadequate support and lack of organisational coordination in team, inadequate support from superiors<br>Resources:<br>Advice from colleagues	Demands:<br>Lack of quiet/break rooms, too few PC workstations	

Abbreviations: HA = Homeless aid, RA = Refugee aid, F = Female, M = Male
